# Supplementary material for: Cryo-electron microscopy structures of capsids and in situ portals of DNA-devoid capsids of human cytomegalovirus
Source: Nat Commun. 2023 Apr 11;14:2025. doi: 10.1038/s41467-023-37779-0 (PMC10090080; doi:10.1038/s41467-023-37779-0)
Supplement: Supplementary file 1 — Supplementary Information [file 41467_2023_37779_MOESM1_ESM.pdf]

Supplementary Information

Cryo-electron microscopy structures of capsids and in situ portals of DNA-devoid capsids of human cytomegalovirus

Zhihai Li, Jingjing Pang, Rongchao Gao, Qingxia Wang, Maoyan Zhang and Xuekui Yu

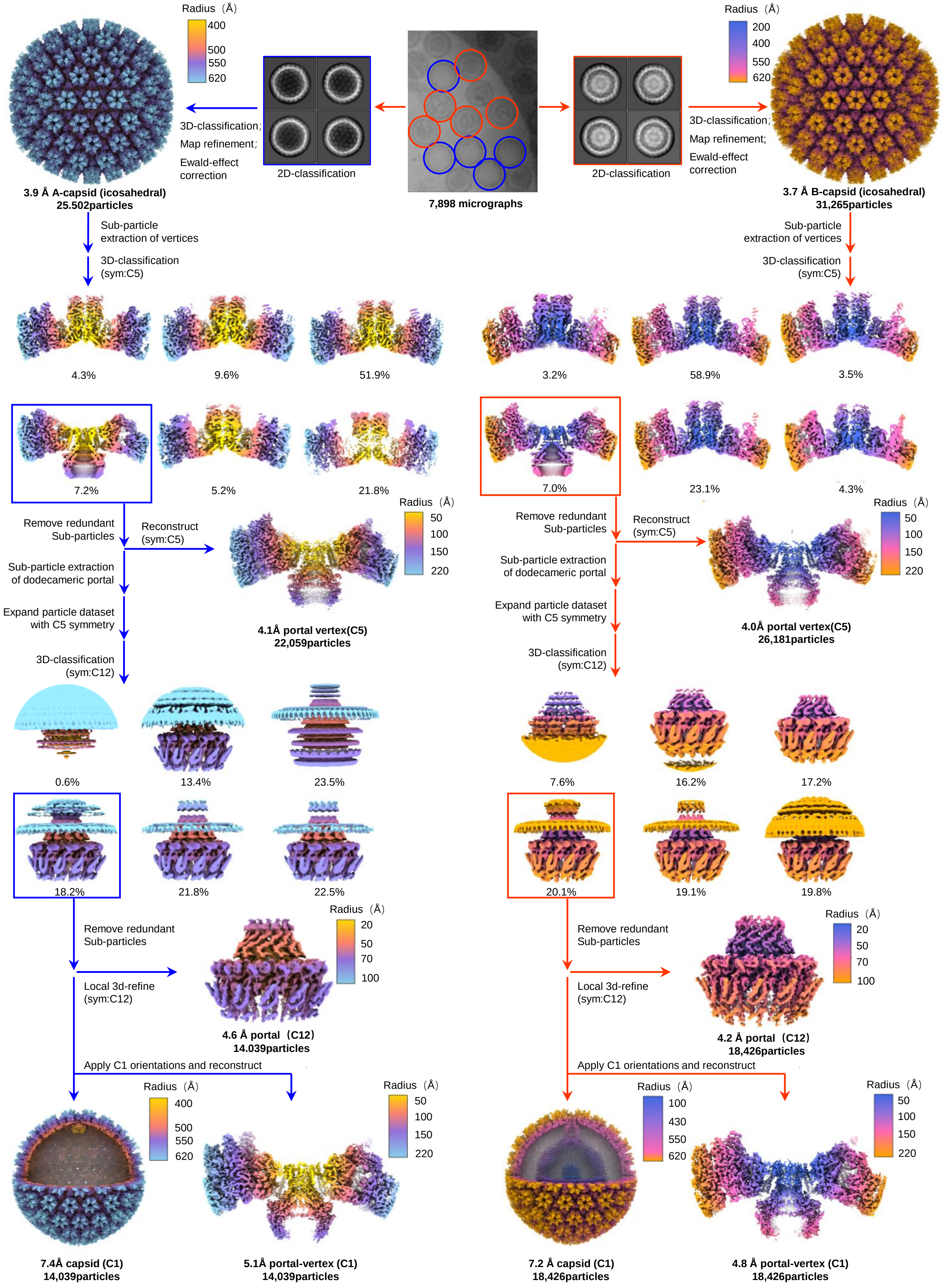

Supplementary Figure 1| Image processing workflow for the capsid and sub-particles from the A- (left) and B-capsids (right)

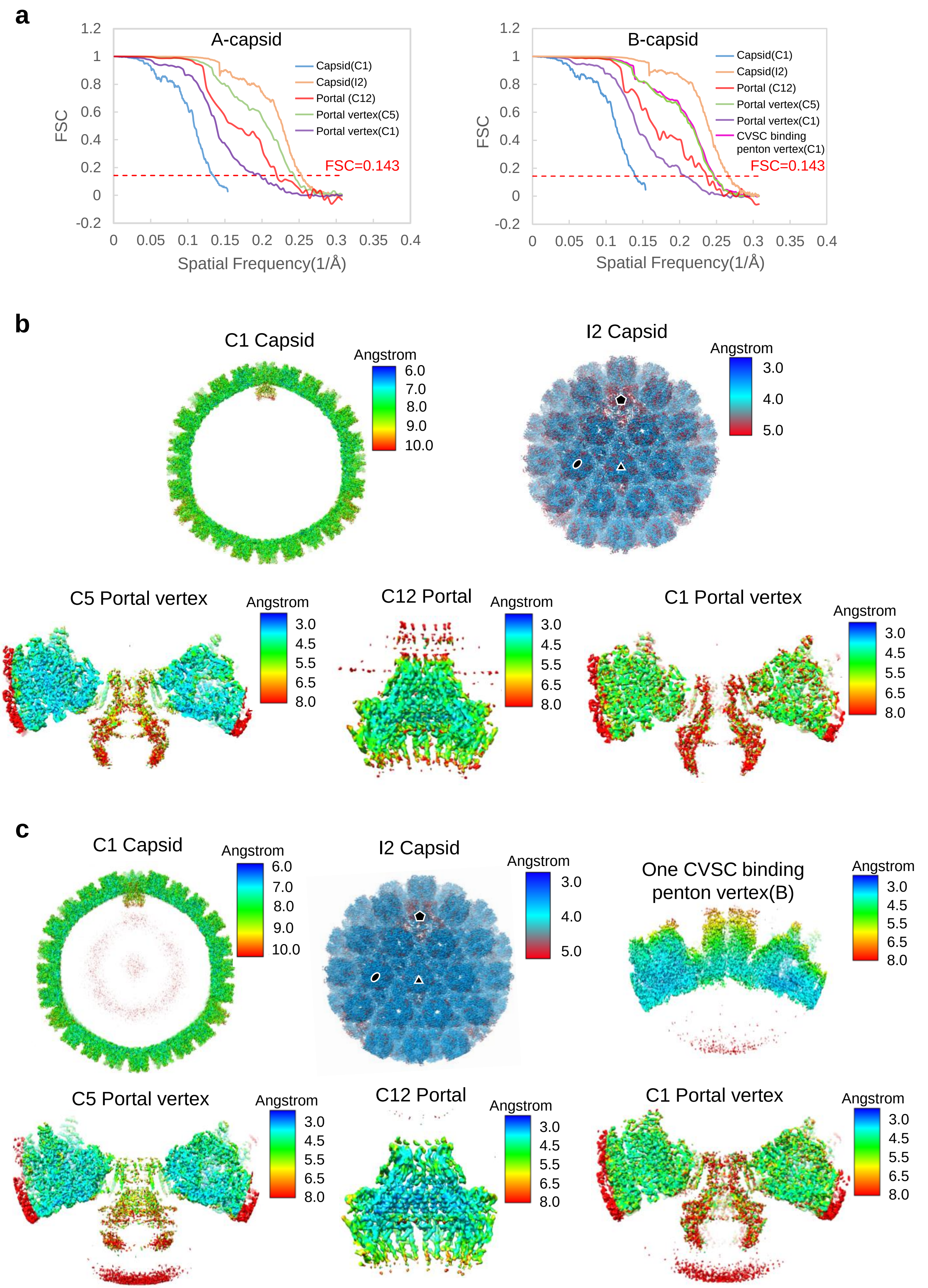
a
A-capsid
FSC
Spatial Frequency(1/Å)
Capsid(C1)
Capsid(I2)
Portal (C12)
Portal vertex(C5)
Portal vertex(C1)
FSC=0.143
B-capsid
CVSC binding
penton vertex(C1)
b
C1 Capsid
I2 Capsid
C5 Portal vertex
C12 Portal
C1 Portal vertex
Angstrom
c
C1 Capsid
I2 Capsid
One CVSC binding
penton vertex(B)
C5 Portal vertex
C12 Portal
C1 Portal vertex
Angstrom

Supplementary Figure 2| Global and local resolution assessment of reconstructions of the capsid and sub-particles from the A- and B-capsids. a, Gold-standard FSC curves of the cryoEM reconstructions of the capsids and subparticles for the A- (**left**) and B-capsids (**right**). **b-c,** Local resolution distributions of density maps of the A- (**b**) and B-capsids (**c**), estimated by ResMap.

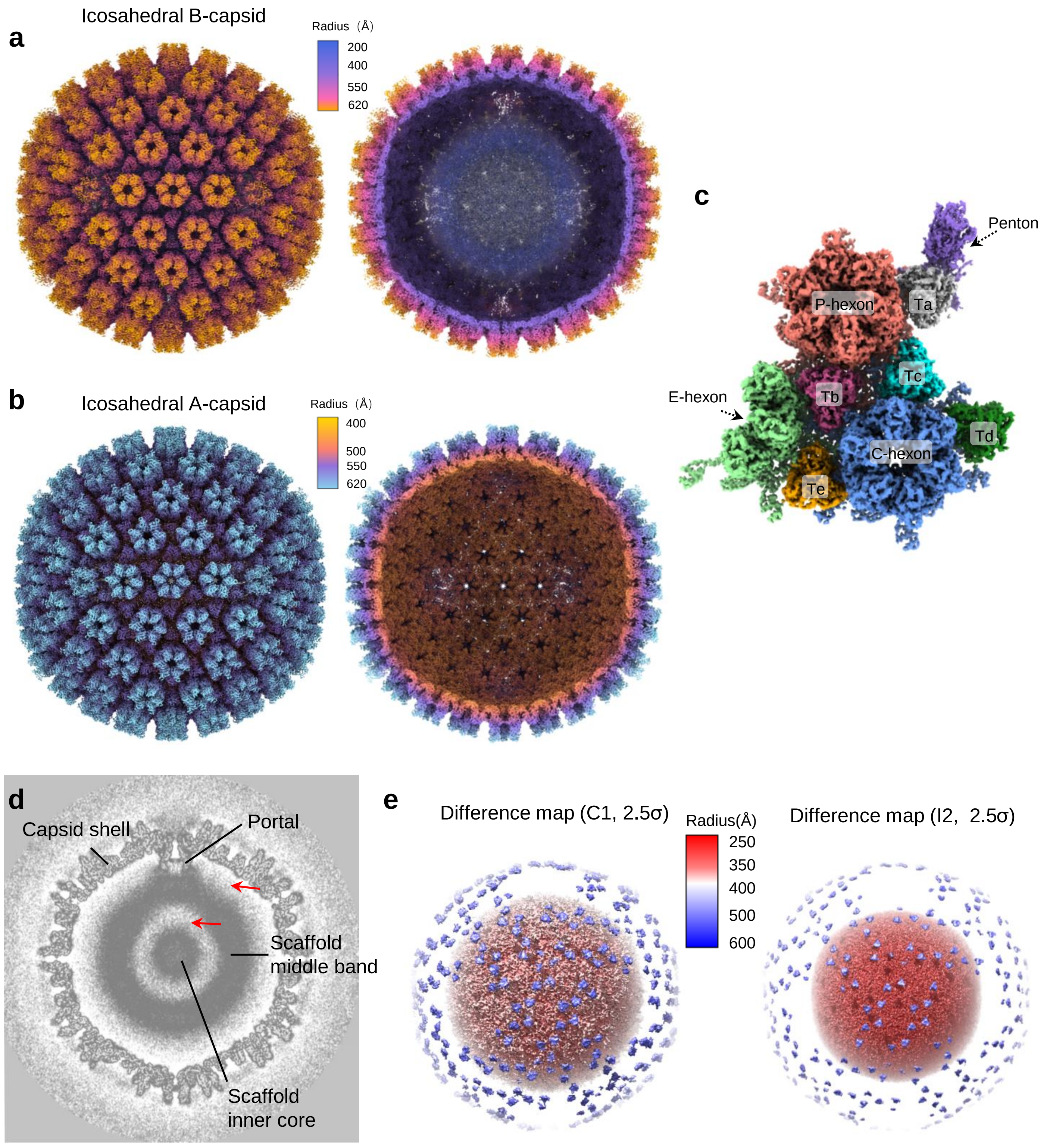

Supplementary Figure 3| Reconstructions of the B- and A-capsids, and difference maps between the B-capsid and A-capsid. a-b, Shaded surface (**left**) and cross-sections (**right**) of the icosahedral B- (**a**) and A-capsids (**b**). The two maps are radially colored, as indicated. **c**, Segmented map of one asymmetric unit of the icosahedral B-capsid, colored by subunit as indicated. **d**, Central slice of the B-capsid reconstruction. Tenuous densities connecting the three layers of the scaffold are indicated by red arrows. **e**, Difference maps calculated by subtracting the asymmetric (**left**) and icosahedral (**right**) reconstructions of the A-capsid from those of the B-capsid. The density maps are radially colored and the color key is indicated.

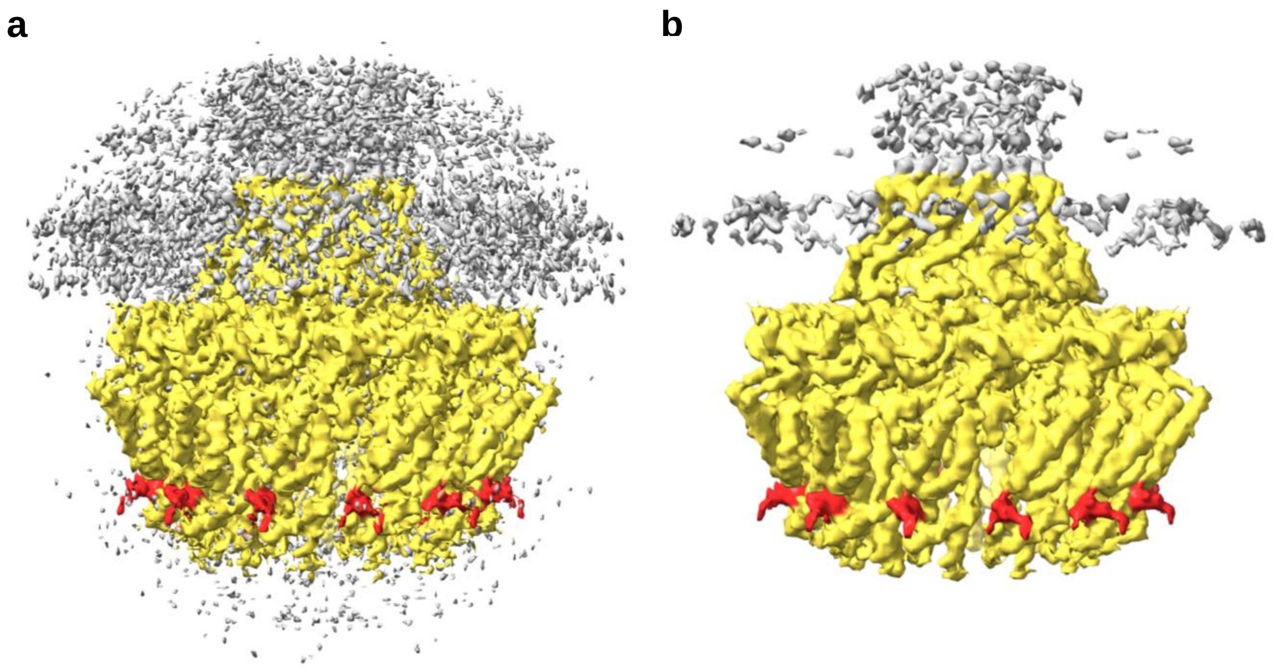

Supplementary Figure 4| Sharpened (a) and unsharpened (b) maps of the C12 portal reconstruction. The portal, the portal-bound scaffold fragments and noised densities are in yellow, red and grey, respectively.

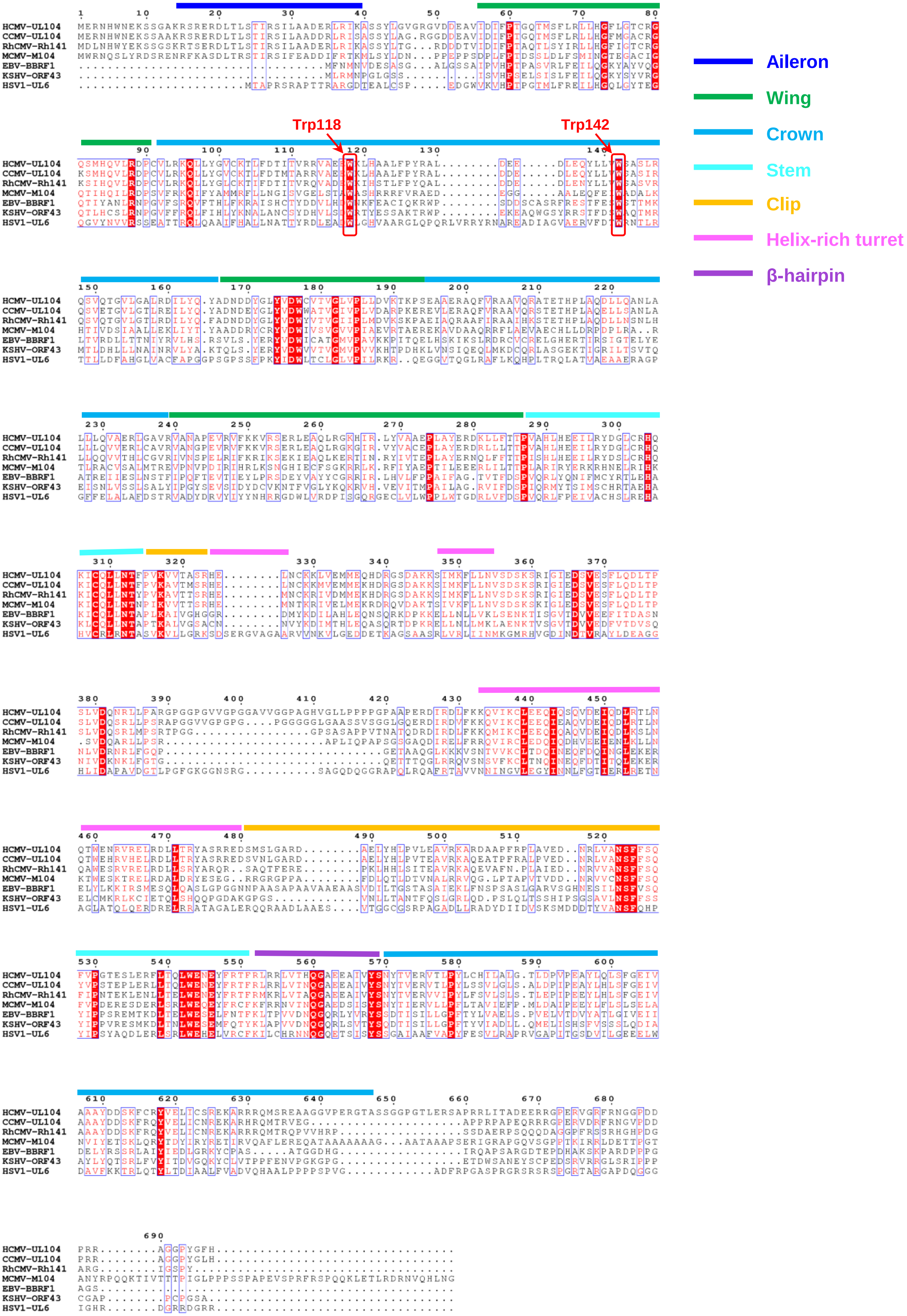

Trp118
Trp142
HCMV-UL104
CCMV-UL104
RhCMV-Rh141
MCMV-M104
EBV-BBRF1
KSHV-ORF43
HSV1-UL6
Aileron
Wing
Crown
Stem
Clip
Helix-rich turret
β-hairpin

Supplementary Figure 5| Sequence alignment of the portal proteins of different herpesviruses, including HCMV, chimpanzee cytomegalovirus (CCMV), rhesus cytomegalovirus (RhCMV), mouse cytomegalovirus (MCMV), Epstein-bar virus (EBV), Kaposi's sarcoma-associated virus (KSHV) and Herpes simplex virus 1 (HSV-1). The two conserved tryptophan residues (Trp118 and Trp142), participating in the interaction with the scaffold, are indicated by red boxes.

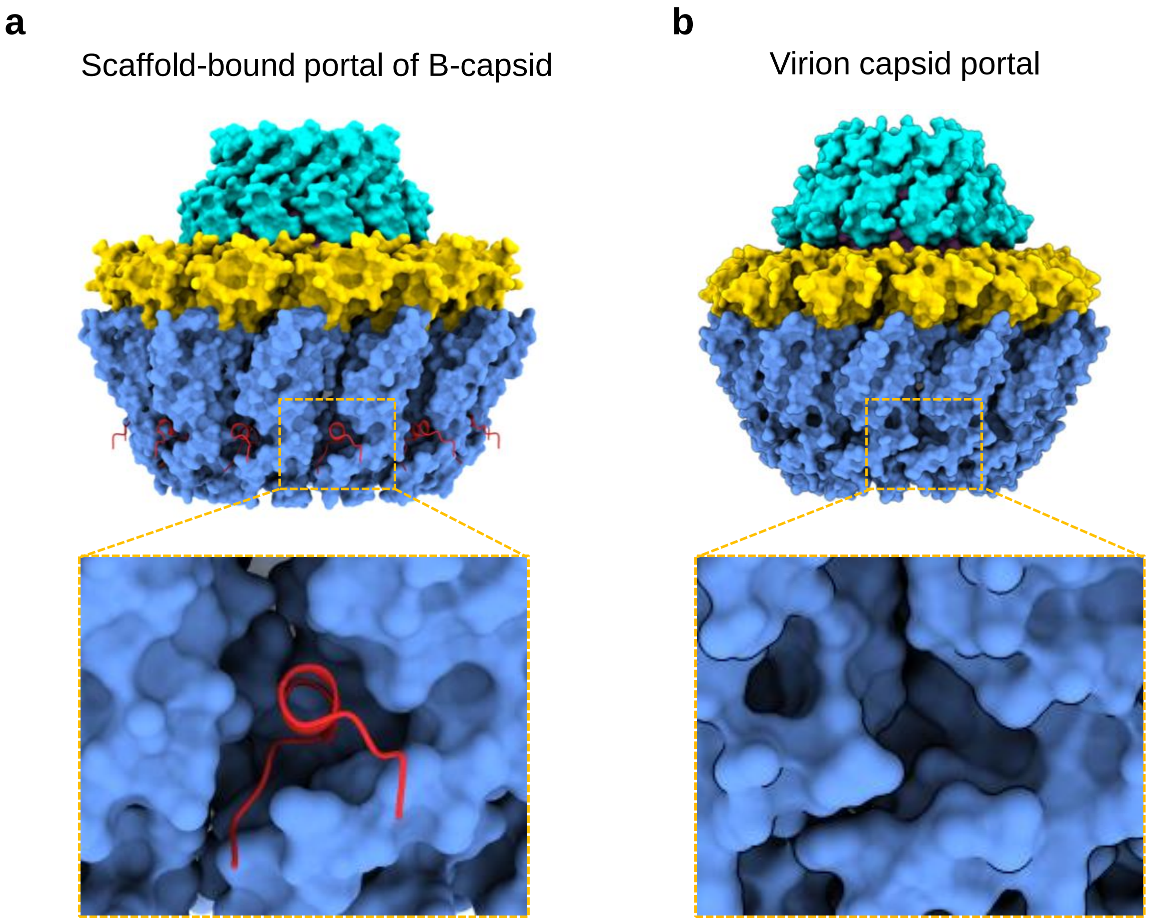

Supplementary Figure 6| Structural comparison of the scaffold binding cavity on portals between B- (a) and virion (b) capsids. Insets are the zoomed-in views of boxed regions, showing the scaffold-binding cavity in B-capsid portal (**a**) is closed off in that of virion capsid (**b**).

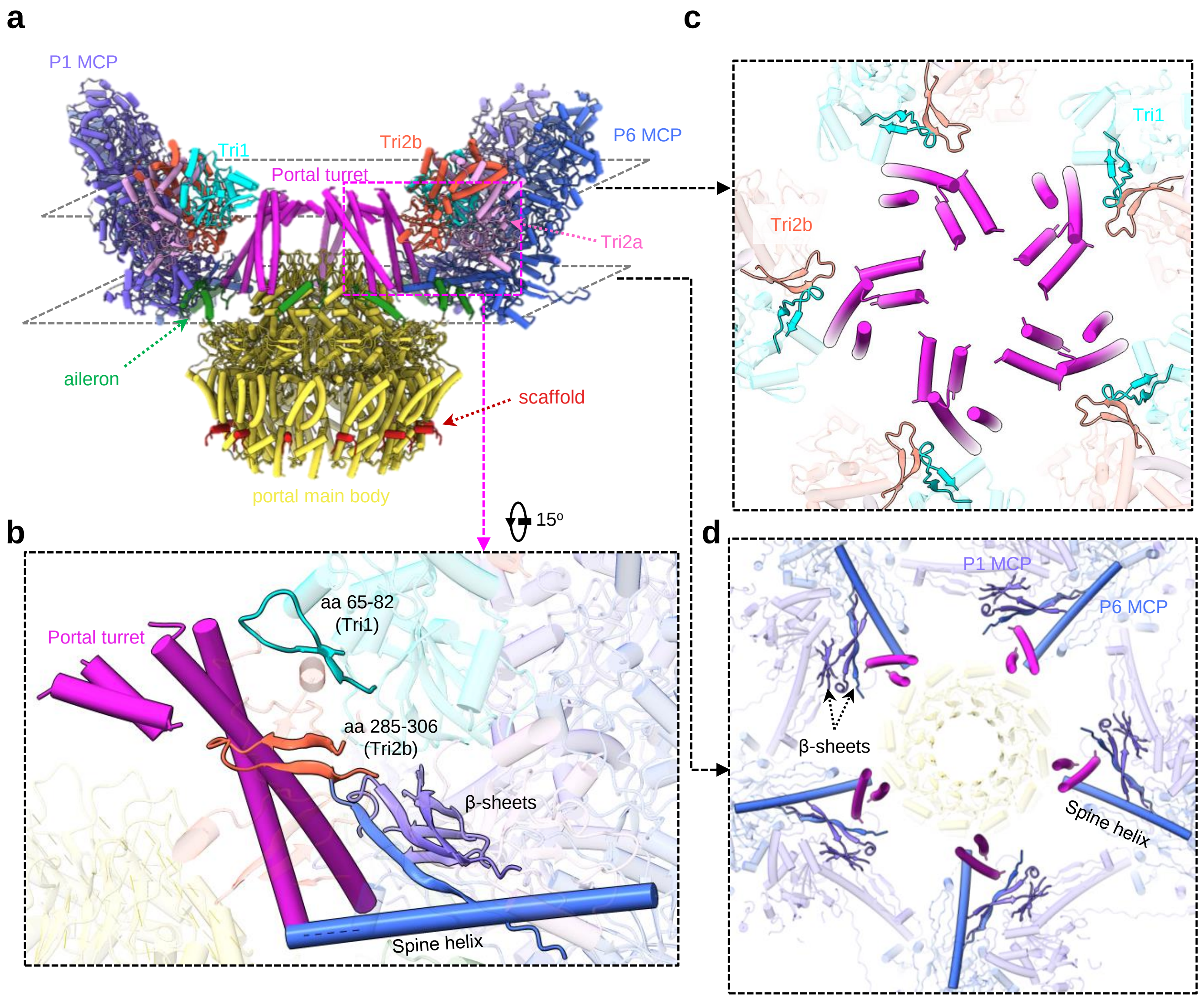

Supplementary Figure 7| Interactions between portal and capsid proteins in DNA-devoid capsid. a, Side view of the portal vertex in the B-capsid. For clarity, only two P1 (purple), two P6 (blue) and two triplexes of Ta (colored by molecule: Tri1, cyan; Tri2a, pink; Tri2b, orange) are shown. The portal is colored by domain: 10-helix anchor, green; turret, magenta; main body, yellow. **b**, Side view of the magenta-boxed region in (**a**), showing the interaction between the portal turret and the capsid proteins. **c-d**, Top views of the two planes of (**a**) as indicated, showing the interactions between the capsid proteins and the upper region (**c**) or lower region (**d**) of the portal turret.

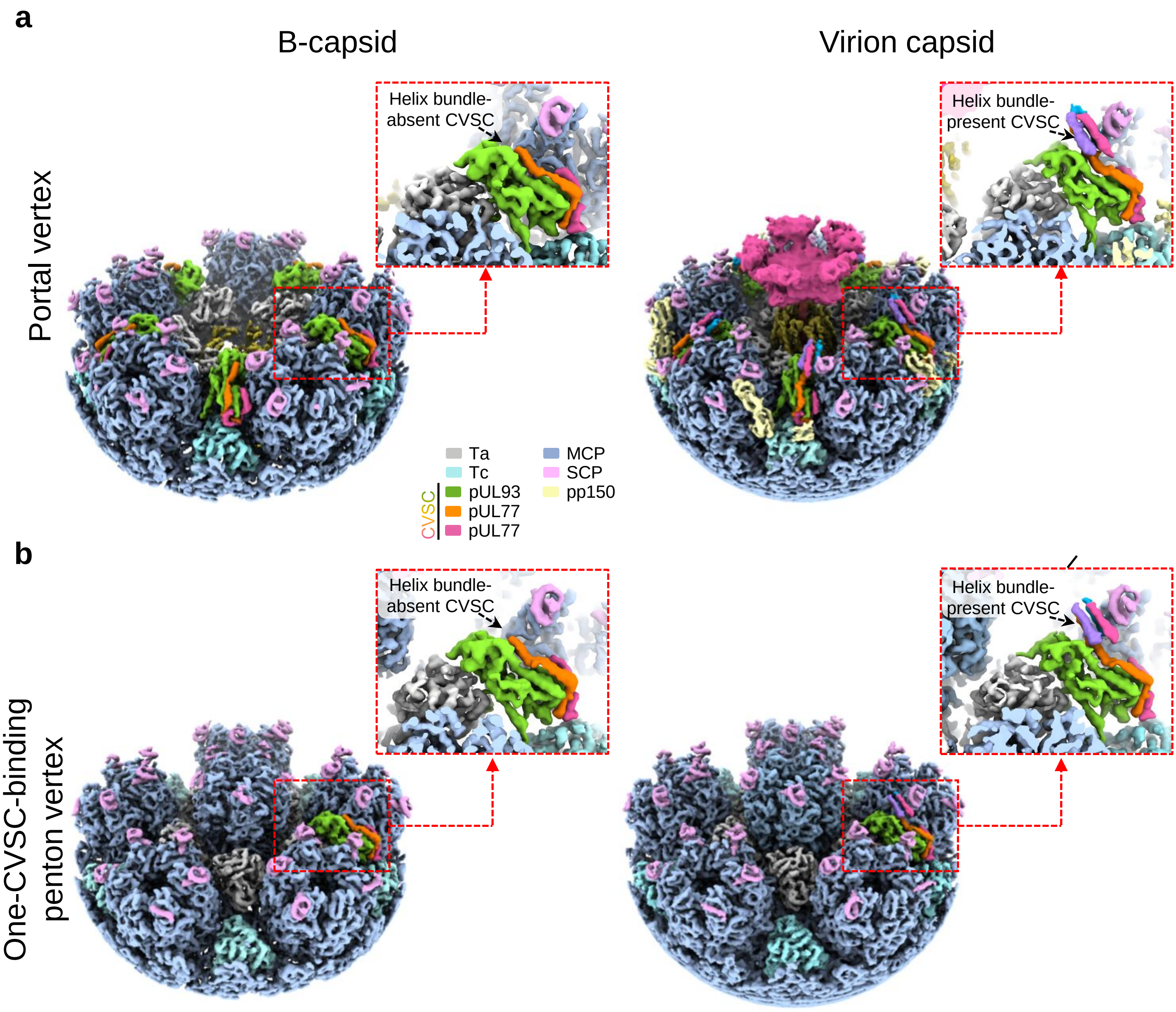

Supplementary Figure 8| Structural comparisons of the peri-portal (a) and peri-penton (b) CVSCs between the B- and virion capsids. Insets are the zoomed-in views of the boxed regions of different vertices, indicating the absence of the featured helix-bundle in the peri-portal and peri-penton CVSCs of the B-capsid.

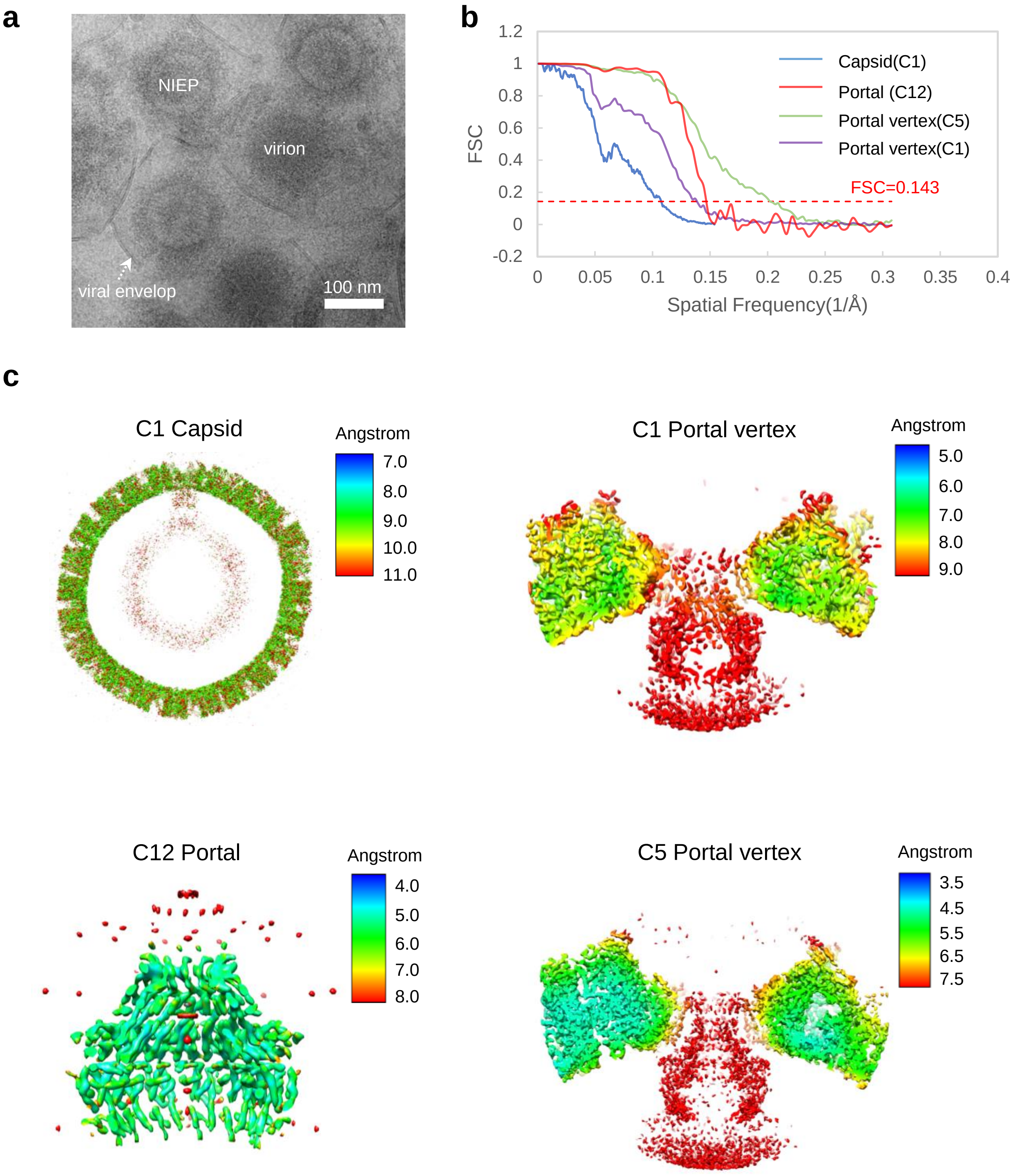

Supplementary Figure 9| A cryoEM micrograph and the resolution assessment of cryoEM maps of HCMV NIEPs. a, A representative micrograph recorded with a 300kv Titan Krios microscope, selected from 10,124 micrographs. **b,** Gold-standard FSC curves of cryoEM reconstructions of the capsid and subparticles of the NIEP capsid. **c,** Local resolution distributions of density maps of the NIEP capsid, estimated by ResMap.

Supplementary Table. 1. Cryo-EM data collection, image processing, and refinement statistics

	A-capsid					B-capsid						NIEPS			
	Icosahedral capsid (EMD: 34986)	C5 portal vertex (EMD: 34691)	C12 portal (EMD: 34692; PDB: 8HEU)	C1 portal vertex (EMD: 34694)	C1 capsid (EMD: 34695)	Icosahedral capsid (EMD: 34699)	C5 portal vertex (EMD: 34696; PDB: 8HEX)	C12 portal (EMD: 34693; PDB: 8HEV)	C1 portal vertex (EMD: 34697)	C1 CVSC binding penton vertex (EMD: 34704; PDB: 8HEY)	C1 capsid (EMD: 34700)	C5 portal vertex (EMD: 34701)	C12 portal (EMD: 34702)	C1 portal vertex (EMD: 34703)	C1 capsid (EMD: 34706)
**Data collection and processing**															
Voltage (kV)	300	300	300	300	300	300	300	300	300	300	300	300	300	300	300
Electron exposure (e–/Å$_2$)	30	30	30	30	30	30	30	30	30	30	30	30	30	30	30
Defocus range (μm)	-1.0 to -2.5	-1.0 to -2.5	-1.0 to -2.5	-1.0 to -2.5	-1.0 to -2.5	-1.0 to -2.5	-1.0 to -2.5	-1.0 to -2.5	-1.0 to -2.5	-1.0 to -2.5	-1.0 to -2.5	-1.0 to -2.5	-1.0 to -2.5	-1.0 to -2.5	-1.0 to -2.5
Pixel size (Å)	1.625	1.625	1.625	1.625	1.625	1.625	1.625	1.625	1.625	1.625	1.625	1.625	1.625	1.625	1.625
Symmetry imposed	I2	C5	C12	C1	C1	I2	C5	C12	C1	C1	C1	C5	C12	C1	C1
Initial particle images (no.)	33,110	22,309	22,059	22,059	22,059	33,480	26,262	26,181	26,181	348,917	26,181	11,635	10,825	10,825	10,825
Final particle images (no.)	25,502	22,059	14,039	14,039	14,039	31,265	26,181	18,426	18,426	40,903	18,426	10,825	8,631	8,631	8,631
Map resolution (Å)	3.9	4.1	4.6	5.1	7.4	3.7	4.0	4.2	4.8	4.1	7.2	5.0	6.9	7.3	9.3
FSC threshold	0.143	0.143	0.143	0.143	0.143	0.143	0.143	0.143	0.143	0.143	0.143	0.143	0.143	0.143	0.143
Map resolution range (Å)	3.0-5.0	3.0-8.0	3.0-8.0	3.0-8.0	6.0-10.0	3.0-5.0	3.0-8.0	3.0-8.0	3.0-8.0	3.0-8.0	6.0-10.0	3.5-7.5	4.0-8.0	5.0-9.0	7.0-11.0
**Refinement**															
Model resolution (Å)	n/a	n/a	4.6	n/a	n/a	n/a	4.1	4.2	n/a	4.1	n/a	n/a	n/a	n/a	n/a
FSC threshold			0.5				0.5	0.5		0.5					
Model resolution range (Å)	n/a	n/a	4.6-50	n/a	n/a	n/a	4.1-50	4.2-50	n/a	4.1-50	n/a	n/a	n/a	n/a	n/a
Map sharpening *B* factor (Å$_2$)	-174	-171	-267	-196	-420	-165	-190	-273	-176	-180	-384	-182	-850	-205	-309
Model composition															
Non-hydrogen atoms	n/a	n/a	40,464	n/a	n/a	n/a	82,236	41,364	n/a	90,224	n/a	n/a	n/a	n/a	n/a
Protein residues			5,004				10,344	5,184		11,348					
Ligands			0				0	0		0					
B factors (Å$_2$)															
Protein	n/a	n/a	128	n/a	n/a	n/a	103	109	n/a	96	n/a	n/a	n/a	n/a	n/a
Ligand															
R.m.s. deviations															
Bond lengths (Å)	n/a	n/a	0.006	n/a	n/a	n/a	0.007	0.007	n/a	0.005	n/a	n/a	n/a	n/a	n/a
Bond angles (°)			1.076				0.982	1.130		0.920					
Validation															
MolProbity score	n/a	n/a	1.80	n/a	n/a	n/a	1.81	1.79	n/a	1.75	n/a	n/a	n/a	n/a	n/a
Clashscore			8.10				7.26	8.55		6.38					
Poor rotamers (%)			0.79				0.53	0.79		0.42					
Ramachandran plot															
Favored (%)	n/a	n/a	94.87	n/a	n/a	n/a	93.79	95.29	n/a	94.00	n/a	n/a	n/a	n/a	n/a
Allowed (%)			5.13				6.21	4.71		5.99					
Disallowed (%)			0.00				0.00	0.00		0.01					
