# Supplementary material for: Cryo-electron microscopy structures of capsids and in situ portals of DNA-devoid capsids of human cytomegalovirus
Source: Nat Commun. 2023 Apr 11;14:2025. doi: 10.1038/s41467-023-37779-0 (PMC10090080; doi:10.1038/s41467-023-37779-0)
Supplement: Supplementary file 3 — Reporting Summary [file 41467_2023_37779_MOESM3_ESM.pdf]

nature portfolio

Corresponding author(s): Xuekui Yu

Last updated by author(s): Mar 24, 2023

Reporting Summary

Nature Portfolio wishes to improve the reproducibility of the work that we publish. This form provides structure for consistency and transparency in reporting. For further information on Nature Portfolio policies, see our Editorial Policies and the Editorial Policy Checklist.

Statistics

For all statistical analyses, confirm that the following items are present in the figure legend, table legend, main text, or Methods section.

n/a	Confirmed	
☒	☐	The exact sample size (*n*) for each experimental group/condition, given as a discrete number and unit of measurement
☐	☒	A statement on whether measurements were taken from distinct samples or whether the same sample was measured repeatedly
☒	☐	The statistical test(s) used AND whether they are one- or two-sided<br>*Only common tests should be described solely by name; describe more complex techniques in the Methods section.*
☒	☐	A description of all covariates tested
☒	☐	A description of any assumptions or corrections, such as tests of normality and adjustment for multiple comparisons
☒	☐	A full description of the statistical parameters including central tendency (e.g. means) or other basic estimates (e.g. regression coefficient) AND variation (e.g. standard deviation) or associated estimates of uncertainty (e.g. confidence intervals)
☒	☐	For null hypothesis testing, the test statistic (e.g. *F*, *t*, *r*) with confidence intervals, effect sizes, degrees of freedom and *P* value noted<br>*Give P values as exact values whenever suitable.*
☒	☐	For Bayesian analysis, information on the choice of priors and Markov chain Monte Carlo settings
☒	☐	For hierarchical and complex designs, identification of the appropriate level for tests and full reporting of outcomes
☒	☐	Estimates of effect sizes (e.g. Cohen's *d*, Pearson's *r*), indicating how they were calculated

Our web collection on statistics for biologists contains articles on many of the points above.

Software and code

Policy information about availability of computer code

Data collection	SerialEM v3.7
Data analysis	Relion v3.0, MotionCor2 v1.2.2, CTFFIND4, PHENIX-1.14, COOT 0.8.9.1, ChimerX v1.3, ResMap v1.1.4

For manuscripts utilizing custom algorithms or software that are central to the research but not yet described in published literature, software must be made available to editors and reviewers. We strongly encourage code deposition in a community repository (e.g. GitHub). See the Nature Portfolio guidelines for submitting code & software for further information.

Data

Policy information about availability of data

All manuscripts must include a data availability statement. This statement should provide the following information, where applicable:

- Accession codes, unique identifiers, or web links for publicly available datasets
- A description of any restrictions on data availability
- For clinical datasets or third party data, please ensure that the statement adheres to our policy

All density maps generated in this study have been deposited in the Electron Microscopy Bank under accession codes EMD-34698 (Icosahedral A-capsid)[https://www.ebi.ac.uk/pdbe/entry/emdb/EMD-34698], EMD-34691 (C5 portal vertex of A-capsid)[https://www.ebi.ac.uk/pdbe/entry/emdb/EMD-34691], EMD-34692 (C12 portal of A-capsid)[https://www.ebi.ac.uk/pdbe/entry/emdb/EMD-34692], EMD-34694 (C1 portal vertex of A-capsid)[https://www.ebi.ac.uk/pdbe/entry/emdb/EMD-34694], EMD-34695 (C1 A-capsid)[https://www.ebi.ac.uk/pdbe/entry/emdb/EMD-34695], EMD-34699 (Icosahedral B-capsid)[https://www.ebi.ac.uk/pdbe/

entry/emdb/EMD-34699], EMD-34696 (C5 portal vertex of B-capsid)[https://www.ebi.ac.uk/pdbe/entry/emdb/EMD-34696], EMD-34693 (C12 portal of B-capsid)[https://www.ebi.ac.uk/pdbe/entry/emdb/EMD-34693], EMD-34697 (C1 portal vertex of B-capsid)[https://www.ebi.ac.uk/pdbe/entry/emdb/EMD-34697], EMD-34704 (C1 CVSC-binding penton vertex of B-capsid)[https://www.ebi.ac.uk/pdbe/entry/emdb/EMD-34704], EMD-34700 (C1 B-capsid)[https://www.ebi.ac.uk/pdbe/entry/emdb/EMD-34700], EMD-34701 (C5 portal vertex of NIEPs capsid)[https://www.ebi.ac.uk/pdbe/entry/emdb/EMD-34701], EMD-34702 (C12 portal of NIEPs capsid)[https://www.ebi.ac.uk/pdbe/entry/emdb/EMD-34702], EMD-34703 (C1 portal vertex of NIEPs capsid)[https://www.ebi.ac.uk/pdbe/entry/emdb/EMD-34703] and EMD-34706 (C1 NIEPs capsid)[https://www.ebi.ac.uk/pdbe/entry/emdb/EMD-34706]. The atomic coordinates generated in this study have been deposited in the Protein Data Bank under accession code 8HEU (C12 portal of A-capsid)[http://doi.org/10.2210/pdb8HEU/pdb], 8HEV (C12 portal binding with scaffold fragments of B-capsid)[http://doi.org/10.2210/pdb8HEV/pdb], 8HEX (C5 portal vertex of B-capsid)[http://doi.org/10.2210/pdb8HEX/pdb] and 8HEY (C1 one CVSC-binding penton vertex)[http://doi.org/10.2210/pdb8HEY/pdb], respectively. The density maps of HCMV virion capsid used in this study are available in the Electron Microscopy Bank under accession codes EMD-31292 (C1 capsid)[https://www.ebi.ac.uk/pdbe/entry/emdb/EMD-31292], EMD-31290 (C1 portal vertex)[https://www.ebi.ac.uk/pdbe/entry/emdb/EMD-31290], EMD-31297 (C5 portal vertex)[https://www.ebi.ac.uk/pdbe/entry/emdb/EMD-31297], EMD-31299 (C6 portal)[https://www.ebi.ac.uk/pdbe/entry/emdb/EMD-31299] and EMD-31295 (C12 portal main body)[https://www.ebi.ac.uk/pdbe/entry/emdb/EMD-31295].

Human research participants

Policy information about studies involving human research participants and Sex and Gender in Research.

Reporting on sex and gender	No human research was included
Population characteristics	No human research was included
Recruitment	No human research was included
Ethics oversight	No human research was included

Note that full information on the approval of the study protocol must also be provided in the manuscript.

Field-specific reporting

Please select the one below that is the best fit for your research. If you are not sure, read the appropriate sections before making your selection.

☒ Life sciences ☐ Behavioural & social sciences ☐ Ecological, evolutionary & environmental sciences

For a reference copy of the document with all sections, see nature.com/documents/nr-reporting-summary-flat.pdf

Life sciences study design

All studies must disclose on these points even when the disclosure is negative.

Sample size	25,502 and 31,265 particles were selected through 2D- and 3D-classifications and used for structure determinations of icosahedral A- and B-capsid, respectively, and their derived subparticles. The data size of cryoEM was determined by the availability of the microscope time and the particle density on the grids.
Data exclusions	Micrographs with poorly fitted CTFs were excluded from further image processing.
Replication	During the sample preparation of A- and B-capsids, we screened different batches of cryo-samples and collected a small data set for each of them. The reproducibility was confirmed in cryo-micrographs, 2D- and 3D-classifications.
Randomization	According to the sample purity and particle distribution on the grid, the best sample grid was used for CryoEM data collection , thus the sample randomization is not relevant to our study; For 3D refinement in Relion v3.0, the particle dataset was randomly divided into two subsets. Otherwise, no randomization was performed.
Blinding	Experimental results did not require any subjective analysis, thus no experiments were preformed with blinding

Reporting for specific materials, systems and methods

We require information from authors about some types of materials, experimental systems and methods used in many studies. Here, indicate whether each material, system or method listed is relevant to your study. If you are not sure if a list item applies to your research, read the appropriate section before selecting a response.

Materials & experimental systems

n/a	Involved in the study	
☒	☐	Antibodies
☐	☒	Eukaryotic cell lines
☒	☐	Palaeontology and archaeology
☒	☐	Animals and other organisms
☒	☐	Clinical data
☒	☐	Dual use research of concern

Methods

n/a	Involved in the study	
☒	☐	ChIP-seq
☒	☐	Flow cytometry
☒	☐	MRI-based neuroimaging

Eukaryotic cell lines

Policy information about cell lines and Sex and Gender in Research

Cell line source(s)	Human fibroblast MRC-5 (ATCC No. CCL-171)
Authentication	No further authentication was performed for commercially available cell lines.
Mycoplasma contamination	No mycoplasma contamination.
Commonly misidentified lines (See ICLAC register)	No commonly misidentified cell lines were used.
